# A New Method for Single-Epoch Ambiguity Resolution with Indoor Pseudolite Positioning

**DOI:** 10.3390/s17040921

**Published:** 2017-04-21

**Authors:** Xin Li, Peng Zhang, Jiming Guo, Jinling Wang, Weining Qiu

**Affiliations:** 1 School of Geodesy and Geomatics, Wuhan University, Wuhan 430079, China; whuxin@hotmail.com (X.L.); jmguo@sgg.whu.edu.cn (J.G.); wnqiu@sgg.whu.edu.cn (W.Q.); 2 Key Laboratory of Precise Engineering and Industry Surveying of National Administration of Surveying, Mapping and Geoinformation, Wuhan University, Wuhan 430079, China; 3 School of Surveying and Spatial Information Systems, The University of New South Wales, 2052 Sydney, Australia; Jinling.wang@unsw.edu.au

**Keywords:** pseudolite positioning, ambiguity resolution, ambiguity function method, improved particle swarm optimization

## Abstract

Ambiguity resolution (AR) is crucial for high-precision indoor pseudolite positioning. Due to the existing characteristics of the pseudolite positioning system, such as the geometry structure of the stationary pseudolite which is consistently invariant, the indoor signal is easy to interrupt and the first order linear truncation error cannot be ignored, and a new AR method based on the idea of the ambiguity function method (AFM) is proposed in this paper. The proposed method is a single-epoch and nonlinear method that is especially well-suited for indoor pseudolite positioning. Considering the very low computational efficiency of conventional AFM, we adopt an improved particle swarm optimization (IPSO) algorithm to search for the best solution in the coordinate domain, and variances of a least squares adjustment is conducted to ensure the reliability of the solving ambiguity. Several experiments, including static and kinematic tests, are conducted to verify the validity of the proposed AR method. Numerical results show that the IPSO significantly improved the computational efficiency of AFM and has a more elaborate search ability compared to the conventional grid searching method. For the indoor pseudolite system, which had an initial approximate coordinate precision better than 0.2 m, the AFM exhibited good performances in both static and kinematic tests. With the corrected ambiguity gained from our proposed method, indoor pseudolite positioning can achieve centimeter-level precision using a low-cost single-frequency software receiver.

## 1. Introduction

Global Navigation Satellite Systems (GNSS) have many advantages, including all-weather use, a high precision, and reliability in an outdoor environment [1]. However, the GNSS signal is susceptible to obstacles, leading to its incompetence for positioning in many constrained conditions. However, the indoor pseudolite system can overcome the fatal defect of the GNSS in an indoor environment to some extent [2,3,4,5]. Currently, famous pseudolite systems, such as Locata [6] and i-Going [7], adopt a complicated clock synchronization mechanism to ensure pseudolite clock synchronization. These systems are costly, but perform well [8,9,10]. In fact, in an indoor environment, the multipath effects are serious, the pseudorange accuracy is poor, and the carrier phase observations are frequently interrupted. The static multi-epoch cumulative observation is meaningless due to the invariant geometry structure of the stationary pseudolite [11,12], and because the linear truncation error cannot be ignored when using the conventional linear estimation method [13,14], making precise pseudolite positioning more challenging, especially for a low-cost pseudolite system.

Similar to GNSS positioning, the key to indoor pseudolite high-precision positioning (cm-level) is the correct resolution of the carrier phase ambiguity [15]. Generally, ambiguity resolution (AR) methods are divided into two categories, that is, a search technique in the ambiguity domain and a search technique in the coordinate domain [16]. For the first category, the float solution and corresponding variance–covariance of the ambiguity are generally gained from the GNSS/pseudolite observations, and the fixed ambiguity solution can then be solved based on the integer least squares (ILS) theory, particularly for the popular LAMBDA method [17,18,19,20,21]. When conducting a static observation with a pseudolite system, unlike the GNSS system, the receiver and pseudolites all maintain a stationary state. Therefore, the geometry structure is invariant during the static observation, leading to the pesudorane and carrier phase observation in each continuous epochs also being invariant (without considering the influence of observation noise and cycle slip). Because of this, if one is using pseudolite observation for static initialization, the equations are correlated with each epoch, and the ambiguities can’t be calculated from the equations, and accumulating static data for the pseudolite system is thus meaningless. To overcome this problem, the Known Point Initialization (KPI) method [22,23], which involves placing the rover station at a fixed point, is usually used. The KPI usually requires a higher precision of the known coordinates when using the LAMBDA method. In many cases, even if the known points reach cm-level precision, it still cannot pass the ambiguity validation (such as Ratio test) and is inconvenient for the mobile carrier without a centering device. Therefore, the single-epoch AR mode is almost impossible for the pseudolite system using the LAMBDA method (without including other extra information such as the initial approximate coordinates), because when the receiver is in a state of movement, it difficult to obtain a relatively high accuracy initial coordinate. Wan et al. [22] conducted the simulation experiment for the pseudolite AR method using LAMBDA with an extended Kalman filter (EKF). Li et al. [23] proposed a new pseudolite AR method using LAMBDA in which the feature is not in need of an initial known coordinate by accumulating the kinematic data, and this exhibited a good performance in the case of no signal interruption and no cycle slip of the carrier phase observations, despite it still being difficult to ensure in an indoor environment.

In this paper, we propose a well-suited AR method for indoor pseudolite precise positioning, which is based on the ambiguity function method (AFM) [24,25,26]. By using an objective function, the best solution in the predefined search space and the possible rover positions can be searched. The conventional grid searching method of AFM requires a considerable amount of computation time [27,28], thereby seriously limiting its application in the AR field. However, with the development of computer technology, many intelligent optimization algorithms have been applied to engineering problems in recent years [29,30,31,32,33,34]. In this study, we adopt an improved particle swarm optimization (IPSO) method to overcome a low computational efficiency. On the other hand, the reliability of AFM depends on the corrected decidedness of the complicated multipeak function [35]. Two ideas can be adopted for improving the reliability of AFM, namely, to increase the ambiguity function value (AFV) of the correct solution through more observations [36] and to reduce the size of the search space to avoid the incorrect solution as much as possible [37,38]. For GNSS positioning, we can accumulate static observations for the easy selection of the correct coordinate candidate to improve the reliability of AFM. As demonstrated above, accumulating static data for the pseudolite system is invalid, and thus, considering the multipeak problem of the AFM for the pseudolite system, it is important to determine a reasonable search space, which can guarantee the accuracy of the initial approximate coordinate, but which can also search for the correct optimal solution as much as possible. In order to further improve the reliability of AFM, we use LS adjustment variances when determining the baseline parameters [39].

The proposed AR method is based on the double differential (DD) model for the pseudolite system. Therefore, the receiver and pseudolite clock errors are eliminated, making the strict clock synchronization unnecessary. In addition to the single-frequency pseudolite receivers, it does not require additional sensors. Therefore, the whole system is relatively low cost. The AFM does not require float ambiguities and a variance–covariance matrix as it is an instantaneous AR technique (enables an AR for every single epoch) and is therefore resistant to cycle slips. A nonlinear AR method can also effectively prevent coordinate linear error effects, which are all quite suited for the pseudolite positioning system. In this paper, the AFM method based on an IPSO will be introduced first, as well as the corresponding strategies to improve the reliability of AFM for the indoor pseudolite system. Then, several experiments, including static and kinematic tests, are used to validate the proposed AR method. Finally, some valuable conclusions will be presented.

## 2. Method Introduction

### 2.1. DD Observation Equation with Pseudolite System

Pseudolite observations include the pseudorange and carrier phase, which can be denoted as $P_{r}^{s}$ and $\phi_{r}^{s}$ respectively, on the receiver–pseudolite pair s–r. The observation equations [40,41] can be formulated as:

(1) $$\begin{array}{l} P_{r}^{s}=\rho_{r}^{s}+cdt_{r}^{s}+e_{r}^{s}\\ \phi_{r}^{s}=\rho_{r}^{s}+cdt_{r}^{s}+\lambda N_{r}^{s}+\varepsilon_{r}^{s} \end{array}$$

where $\rho_{r}^{s}$ is the receiver–pseudolite range, $dt_{r}^{s}$ is the clock errors, $N_{r}^{s}$ is the carrier phase ambiguity, c is the speed of light, λ is the wavelength of the corresponding carrier-phase, and $e_{r}^{s}$ and $\varepsilon_{r}^{s}$ are the remaining error terms.

From Equation (1), the structure of the pseudorange and carrier phase observation is similar, with the latter containing an ambiguous term. This structure implies that as long as the ambiguity can be resolved correctly, the carrier phase observation will be transformed to a high-precision receiver–pseudolite pseudorange.

Equation (1) includes two clock bias terms. We usually adopt the classical DD model to eliminate them. The DD observations use simultaneous observations from two receivers and two pseudolites, which can be described as:

(2) $$\begin{array}{l} P_{qr}^{ls}=\rho_{qr}^{ls}+e_{qr}^{ls}\\ \phi_{qr}^{ls}=\rho_{qr}^{ls}+\lambda N_{qr}^{ls}+\varepsilon_{qr}^{ls} \end{array}$$

where $\rho_{qr}^{ls}=\rho_{r}^{s}-\rho_{r}^{l}-(\rho_{q}^{s}-\rho_{q}^{l})$, $P_{qr}^{ls}$ and $\phi_{qr}^{ls}$, respectively, denote the DD pseudorange and carrier phase observation, and $N_{qr}^{ls}$ denotes the DD carrier phase ambiguity.

### 2.2. Ambiguity Function Method Overview

The AFM does not explicitly solve the ambiguity parameter using the LS or extended Kalman filter (EKF) estimation, but searches for the best solution of the rover coordinates in a predefined search space; thus, it is a coordinate-based method. The definition of AFM [35] corresponding to single-frequency and single-epoch carrier phase observations is given as follows:

(3) $$\mathrm{AFV}(X_{c},Y_{c},Z_{c})=\frac{1}{n}\sum_{i=1}^{n}\cos2\pi (\phi_{obs}^{ls}[E1|E2]-\phi_{cal}^{ls}[E1|X_{c},Y_{c},Z_{c}])$$

where AFV denotes the Ambiguity Function Value [36], *E*1 denotes the base station, *E*2 is the rover station, $\left(X_{c},Y_{c},Z_{c}\right)$ is the search candidate coordinate of the rover, $\phi_{obs}^{ls}$ is the DD observation, and $\phi_{cal}^{ls}$ is the calculated DD observation according to the candidate points. If the position of the candidate $\left(X_{c},Y_{c},Z_{c}\right)$ is similar to *E*2, because of the integer characteristic of the DD ambiguity, the cosine function difference value between $\phi_{obs}^{ls}$ and $\phi_{cal}^{ls}$ should be equal to one. Considering the noise and the multipath effect of the pseudolite carrier observation, the corresponding AFV is close to one.

If we obtain the approximate coordinates of the rover station in some way, a search volume centered on the approximate rover position can be constructed, to maximize the assessment of the candidates of the AFV in a predefined search volume, containing possible rover positions.

However, the AFM has two disadvantages. First, the computational efficiency of the conventional grid search method is very low. Suppose the search space is 1 m × 1 m × 1 m, and the corresponding search step is 1 mm, this would require 10^9^ individual search points for a single observation. Obviously, a large calculation requirement leads to a low computational efficiency. The AFM is also a multipeak function, which means that there may be more than one point that satisfies the AFV that is close to one. With the observation noise, as well as the effect of unmolded errors, the selected point with the maximum AFV value may be not the best solution for the rover station, especially in a large search space. These two defects have been restricting the application of AFM, despite its many advantages, such as its single-epoch and nonlinear AR capacity. In the next section, a modified AFM with an indoor pseudolite system will be introduced in order to address these present situations.

### 2.3. Improved Ambiguity Function Method

#### 2.3.1. Extended AFM with IPSO for Improving Efficiency

Particle swarm optimization (PSO) is a computational method that optimizes a problem by iteratively trying to improve a candidate solution with regard to a given fitness function. This method not only has a strong global optimization ability, but is also in need of fewer adjustment parameters and has a fast calculation speed. This method has been widely used in function optimization, fuzzy control, and other engineering fields [42,43,44]. In this section, we first give the mathematical model of the standard PSO algorithm. An improved IPSO with a superior performance is then introduced. Finally, the entire calculation process of the ambiguity function method based on the IPSO algorithm is presented.

With an *N*-dimensional search space, the total population size is *n*, assuming $x_{i}=(\begin{array}{cccc} x_{i1} & x_{i2} & \cdots & x_{iN} \end{array}),u_{i}=(\begin{array}{cccc} v_{i1} & v_{i2} & \cdots & v_{iN} \end{array})$, which can be denoted as the position vector and velocity vector of the particle *i*. Its current optimal position is denoted as $pBest_{i}$, and the population current optimal position is denoted as gBest. The PSO algorithm can be described as:

(4) $$\begin{array}{l} v_{in}^{\left(k+1\right)}=\omega v_{in}^{\left(k\right)}+c_{1}r_{1}(pBest_{in}^{\left(k\right)}-x_{in}^{\left(k\right)})+c_{2}r_{2}(gBest_{in}^{\left(k\right)}-x_{in}^{\left(k\right)})\\ x_{in}^{\left(k+1\right)}=x_{in}^{\left(k\right)}+v_{in}^{\left(k+1\right)} \end{array}$$

where $\begin{array}{cccc} i=1, & 2, & \cdots & n \end{array}$; $\begin{array}{cccc} d=1, & 2, & \cdots & N \end{array}$; *ω* denotes the inertia weight; $c_{1}$ and $c_{2}$ are two positive constants, namely, the cognitive learning rate and the social learning rate, respectively; and $r_{1}$ and $r_{2}$ are the random functions in the range [0, 1].

First, the PSO algorithm initializes a group of random particles, before finding the $pBest_{i}$ and gBest according to the fitness function. For each iteration, the particles update their position and speed using these two optimal values based on Equation (4) and continuously approximate the optimal solution, until certain conditions are met to find the optimal solution.

However, in the practical application of PSO, due to the fast convergence speed, the population diversity decreases rapidly, causing prematurity and falling into a local optimum. A number of studies have been conducted to improve its performance [45,46,47]. In this study, drawing on the idea of the genetic variation method, we introduce an improved PSO algorithm (IPSO). First, the population is divided into three groups, that is, the optimal, suboptimal, and poor population according to the fitness function values, expressed as $\left(S_{1},S_{2},S_{3}\right)$. The corresponding population size is *s*. For the optimal population ($S_{1}$), a Gauss mutation [48] is made in the particle evolution process:

(5) $$x_{id}^{k+1}=x_{id}^{k}+Gauss(0,1)\cdot x_{id}^{k}$$

where Gauss(0,1) denotes the random number that follows a Gauss distribution.

The specific calculation steps of the AFM based on the IPSO algorithm are as follows:

(1) Parameter setting: n=60, s=20, c1=c2=0.5, ω=0.8.
(2) In the search space with a given coordinate domain, generate 60 initial particles at a random distribution (include position and speed). According to Equation (3), calculate the fitness function value of every particle and make a fitness evaluation. The larger the AFV is, the higher the fitness value.
(3) Update the $pBest_{i}$ and gBest based on the result of the fitness evaluation above.
(4) Update the position and speed for every particle according to Equation (4).
(5) Based on the fitness function value, divide the population into three groups, namely, the optimal, suboptimal, and poor population $\left(S_{1},S_{2},S_{3}\right)$. According to Equation (5), make a Gauss mutation for $S_{1}$.
(6) If the global optimal value or its corresponding fitness function value difference between the last two iterations is less than a certain threshold, exit the iteration; otherwise, repeat steps (2) to (5).

#### 2.3.2. Strategies for Improving AFM Reliability

When using AFM, the pre-given size of the search space is generally determined by the precision of the rover station approximate coordinate. When a high-precision approximate coordinate is known, it requires not only a smaller search space, but also a higher search efficiency. Less observations can yield a relatively good result, because false candidates can be excluded in AFM decision-making in a relatively small search space. Thus, for the pseudolite system, the key to improving AFM reliability is the reasonable determination of the rover station approximate coordinate, including the procedure of first-time Known Point Initialization (KPI) and receiver kinematic movement.

For GNSS positioning, the approximate coordinate of the rover station on the initial observation epoch can usually be determined from the result of code-based DGNSS positioning, but for the pseudolite system, because the indoor observation space is small, the relative accuracy of the pseudorange is poor, leading to its incompetence in precise positioning, as well as the invalidation of the static simulative observation. Therefore, we generally adopt the KPI method to solve the carrier phase ambiguity. Theoretically, the higher the accuracy of the approximate coordinate, the higher the success rate for obtaining the correct ambiguity. When using the integer rounding (IR) or LAMBDA method, incorrectly assigning a cm-level to the accuracy of the approximate coordinates, while the true accuracy is much worse than a cm-level, if without some extra algorithm (such as Gauss-Newton least-squares iteration) or strategy (such as search strategy) being used before LAMBDA is applied, it is difficult to ensure that the correct solution can be obtained. However, the proposed AR method is a search procedure based on the coordinate domain, which means that it may not need a pre-given approximate coordinate with a very high precision. As long as the best coordinate candidate is within the pre-given search region, it can still search for the true position of the rover station. The accuracy requirement of the approximate coordinate on the initial observation epoch will be discussed in detail in the experiment.

For the receiver movement, determining the rover station approximate coordinate is important when using our proposed single-epoch AR method with the aim of focusing on the pseudolite system. In this paper, several methods are introduced for the approximate coordinate determination during receiver moving status: (1) directly taking the positioning result of the previous epoch as the approximate coordinate of the current epoch; (2) selecting at least three pseudolites with continuous observation and using LS to estimate the approximate coordinate; (3) and receiver speed estimation via high-precision carrier phase observation to update the rover approximate coordinate [49,50,51]. Among these methods, the first one is well-suited for a high sampling rate and low dynamic receiver. The second and third methods are relatively complicated, but can obtain a higher approximate coordinate accuracy. In practical data processing, we can select one reasonable method based on the receiver moving status.

To further improve the reliability of the proposed AR method, the final determined coordinate solution of the rover station will be validated by variances of an LS adjustment. With the determined coordinate solution, the DD ambiguity can be obtained through the IR method, the baseline solution and corresponding variances information can be estimated using the LS method, and the determined coordinate can thus be evaluated using variance information. It’s worth mentioning that the coordinate linearization error is neglected in this process.

## 3. Results and Analysis

### 3.1. Experimental Platform

**Figure 1:**
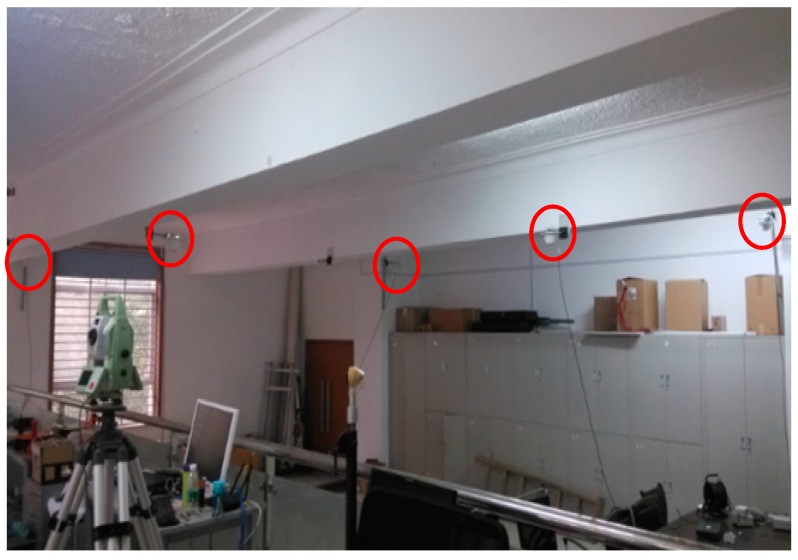
Distribution of pseudolite antennas.

In the experiment, the pseudolite model employed is GSG-L1. This model generates a single L1 carrier and modulates the C/A code. The difference between the transmitted pseudolite signal and the standard GPS L1 signal is: the data content follows a standard GPS sub-frame structure, but the transmitted data currently do not include valid pseudolite positions and clock parameters. Both the base and rover stations choose Universal Software Radio Peripheral (USRP) as the front end of the software receiver. USRP is intended to be a comparatively inexpensive hardware platform for software radio. With the appropriate daughter board configuration, USRP can capture the entire GNSS family of signals. In this system, the DBSRX daughterboard, which can handle signals from 800 MHz to 2.4 GHz, is assembled in each USRP. The pseudolite positioning system was set up in a large room (10 × 7 × 4 m^3^) with five pseudolites mounted on the ceiling, as shown in Figure 1. The locations of the pseudolites are indicated by red circles. The 3-D coordinates of the five pseudolites have been precisely surveyed by a total station using an indoor-independent coordinate system.

Data processing adopts PLRTK V1.2 software, which was independently developed using a standard C language by the Wuhan University team. The computer processor used in our experiment is “Intel(R) Pentium(R) CPU G620@2.60 GHz” and the RAM is 4.0 G.

### 3.2. Performance Analysis of the Proposed AR Method with Pseudolite System

Several experiments including both static and kinematic tests were conducted to verify the validity of the proposed AR method.

The base station receiver antenna is accurately placed at a fixed point in the laboratory, and the rover station was located approximately 0.6 m away from the base station. Because it lacks a strict centering device, the rover station coordinate can only be approximately pre-given, namely, 0.6, 0.6, and 0.01 m, within a positioning error of 0.03 m. Static observation was performed for about half a minute. The data sampling interval is 0.1 s. Five pseudolite signals can be captured without interruption during the experiment.

**Figure 2:**
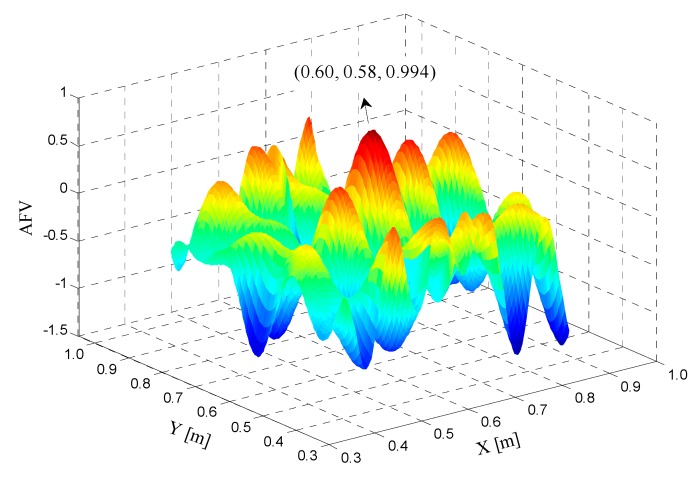
2-D contour map of pseudolite AFV for one epoch.

AFM is performed with a conventional grid search method by using the data of the first epoch carrier phase observations. The initial search approximate coordinate is given as 0.6, 0.6, and 0.01 m, the search range is 0.3 m × 0.3 m, and the search step is 0.005 m, consuming about 0.43 s. Figure 2 shows the 2-D contour map of pseudolite AFV for this epoch. Multiple AFV peaks can be observed in the corresponding search regions. The highest peak corresponds to an AFV value of 0.994, which is close to theoretical value of one. The corresponding optimal coordinates are 0.60 m and 0.58 m, which are within the centering error of the rover station.

**Figure 3:**
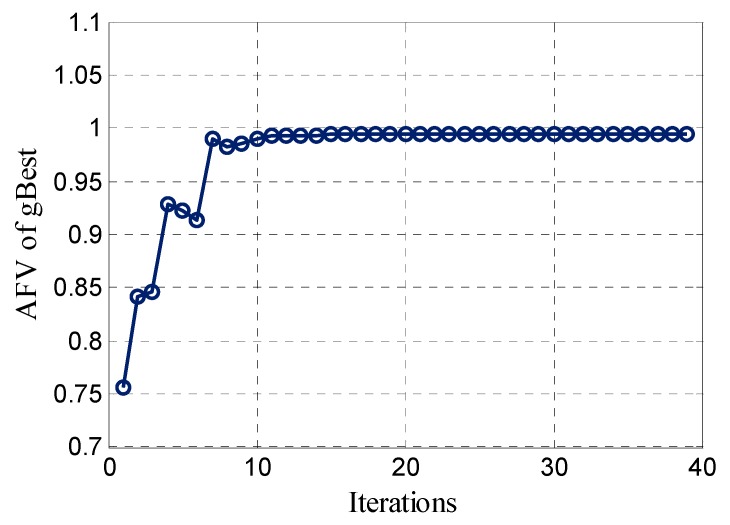
Evolution of global optimal particle AFV using the IPSO method.

The IPSO search method was conducted using the same data as above, and the particle convergence threshold is set to 0.001. Figure 3 shows the evolution of the AFV of the global optimal particle (*gBest*). In the initial state, the particles are generated randomly, and the corresponding AFV of *gBest* is only 0.76, with the continuous positioning and speed update of *pBest* (personal optimal particle) and *gBest*. The homoplasy of particle evolution becomes increasingly apparent until the evolution times reach 38, indicating that the IPSO algorithm satisfies the convergence condition. The total time consumed is 0.034 s.

**Figure 4:**
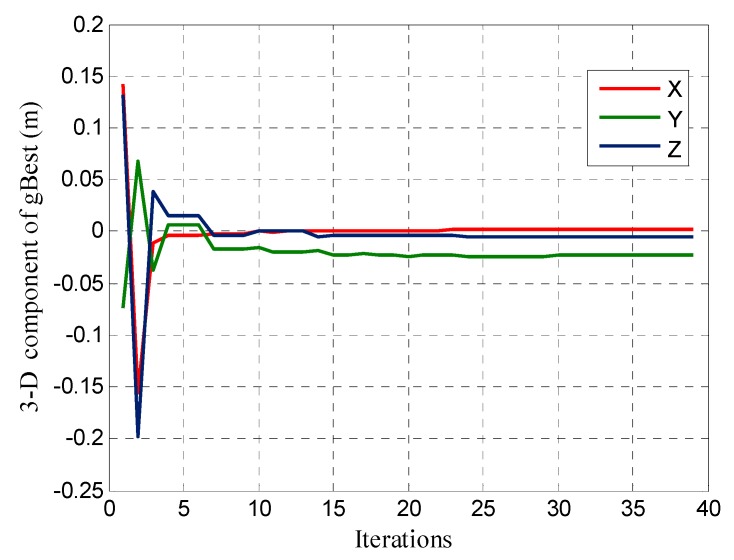
Evolution of the global optimal particle 3-D coordinate component.

Figure 4 shows the evolution of the 3-D coordinates of the global optimal particles (*gBest*), where the 3-D component value is the difference with the initial pre-given coordinates. Compared with Figure 3, we can see that the fitness of *gBest* is smaller, and the difference between the *gBest* position and the actual position is greater. In the gradual convergence process of the 3-D coordinate components of *gBest*, in the early stage of particle evolution, the convergence speed is very fast and the latter is relatively stable. When the AFV difference between the two continuous iterations is less than 1 mm, the IPSO search is complete, indicating that when using the IPSO algorithm to make a coordinate domain search, the search resolution can theoretically reach a submillimeter level.

**Figure 5:**
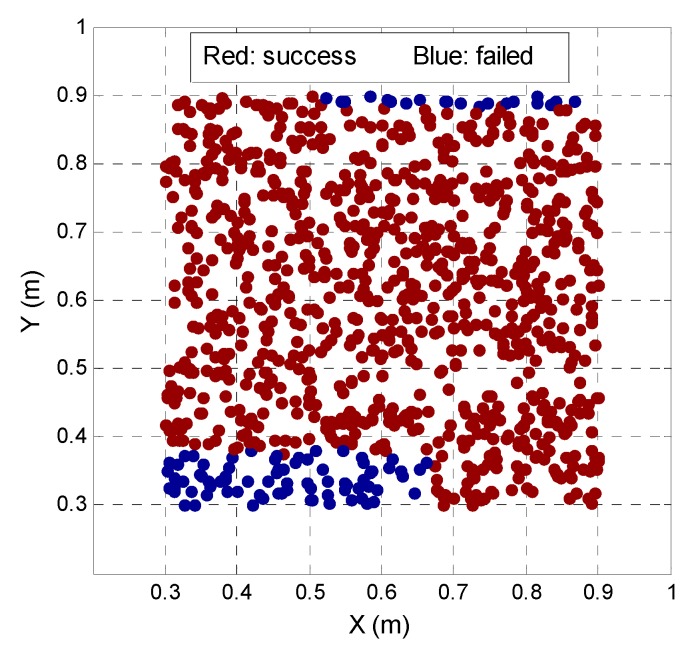
AFM reliability with various preliminary coordinates.

The multi-peak characteristic of the ambiguity function, particularly in the case of less observation, often makes it difficult to absolutely ensure that the highest peak in the search area corresponds to the true optimal coordinates, especially when the search range is too large. In fact, the AFM search range is determined by the accuracy of the initial approximate coordinate. The higher the accuracy, the smaller the search range needed and the higher the reliability of the searched optimal coordinates. The relationship between the AFM reliability and accuracy of the initial approximate coordinate in an indoor environment was verified by randomly generating 1000 points in the 0.3 m × 0.3 m region around the point (0.6, 0.6 and 0.01). Every point is taken as the initial approximate coordinate to conduct an IPSO search using the same data. The search range is set to 0.3 m × 0.3 m. Figure 5 shows the AFM search results for the 1000 points corresponding to plane 2-D scatter distribution, and the 2-D coordinate of every point (both red and blue point) can be obtained from the x-axis and y-axis value. The red points denote that the search optimal solution and the corresponding DD ambiguity is correct, while the blue point is the opposite. From Figure 5, we can see that the blue point appears in the 2-D region far from the initial coordinates, within a 0.2 m × 0.2 m region around the true position, and all points are taken as the approximate coordinate that can search the correct DD ambiguity, indicating that when the accuracy of the approximate coordinate is better than 0.2 m, the AFM exhibits a high reliability and performance for an indoor pseudolite system.

To verify the conclusion that the AFM method is not in need of a very high accurate pre-given initial coordinate compared with the LAMBDA method, we carry out the comparison experiment to demonstrate the influence of initial coordinate bias (ICB) on the proposed AR method and LAMBDA method. The comparison experiment is conducted by using the above data of the first epoch carrier phase observations. Table 1 shows the results of the influence of ICB on the two different AR methods. When the ICB reaches the dm-level, the LAMBDA fails to obtain the correct ambiguity resolution (cannot pass the Ratio validation), while the proposed AR method is still competent, although at the expense of a lower computational efficiency.

In fact, one apparent difference when using LAMBDA and AFM is that, usually when adopting the LAMBDA method, only one pre-given initial coordinate will be used; however, when adopting AFM, many possible initial coordinates within a pre-defined search region will be used to decide the best coordinate by a certain criterion. Therefore, we can say that the search procedure is one strategy, not an AR method, and we can also apply this search strategy when using the LAMBDA method. For example, using many possible initial coordinates within a pre-defined search region, we can decide on one best candidate coordinate which has a maximum corresponding ratio value, and thus, even if the accuracy of a pre-given initial coordinate is not sufficiently high, it may still search for the true position of the rover station and obtain the correct ambiguity resolution.

Considering the high computational efficiency and reliability of LAMBDA, we can further improve our data processing strategy when conducting the Known Point Initialization (KPI) for the pseudolite system. The LAMBDA method can initially be used preferentially, if passing the ratio validation, and the correct ambiguity will be obtained, meaning that the pre-given initial coordinate has a relatively high accuracy and the search strategy does not need to be adopted; after all, when conducting the search strategy, it inevitably wastes unnecessary computing time. If this step fails to obtain the correct ambiguity, meaning the accuracy of the pre-given initial coordinate may be relatively poor, then the search strategy will be used. In this step, both the proposed method and the ‘LAMBDA + search strategy’ model are theoretically feasible, although considering the computational efficiency, the proposed method may have a certain advantage, and considering the reliability, ‘LAMBDA + search strategy’ model may have a certain advantage. This is what we expected to conclude, and in further work, we will pursue an in-depth study on this topic. In this paper, we select an alternative method, namely our proposed method, to deal with the situation when the initial coordinate accuracy is not very high.

#### 3.2.1. Static Test

The above static observation data were processed epoch-by-epoch using our proposed AR method. The initial approximate coordinates of the first-time epoch were pre-given as 0.6, 0.6, and 0.01 m, and the subsequent epochs adopt the first method introduced in Section 2.3.2. The corresponding search space is set to 0.2 m × 0.2 m × 0.2 m. The traditional grid search is carried out at the same time, to highlight the superiority of the IPSO search method. The corresponding search step is set to 0.005 m.

**Figure 6:**
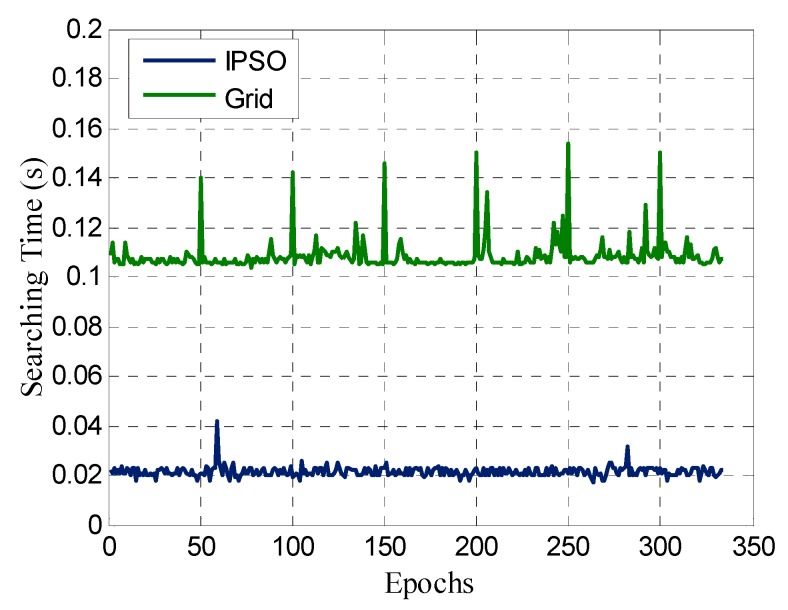
AFM searching time with IPSO and traditional grid methods.

Figure 6 shows the AFM efficiency for two different search methods over the entire observation period. The IPSO method is more efficient than the traditional grid search by an order of magnitude. Compared with the traditional mechanical grid search, IPSO is a new intelligent search method with a high computational efficiency. In fact, the grid search time is closely related to the search space and search step. Table 2 shows the average efficiency of the two search methods for different search spaces and steps. As the search space increases, the efficiency of the grid search decreases rapidly, while the efficiency of the IPSO method was not greatly affected. When the search step is 0.2 m and the search step is set to 0.001 m, the traditional grid search completely loses the search function because of computer memory overflow, while the IPSO is still able to maintain a high computational efficiency.

**Figure 7:**
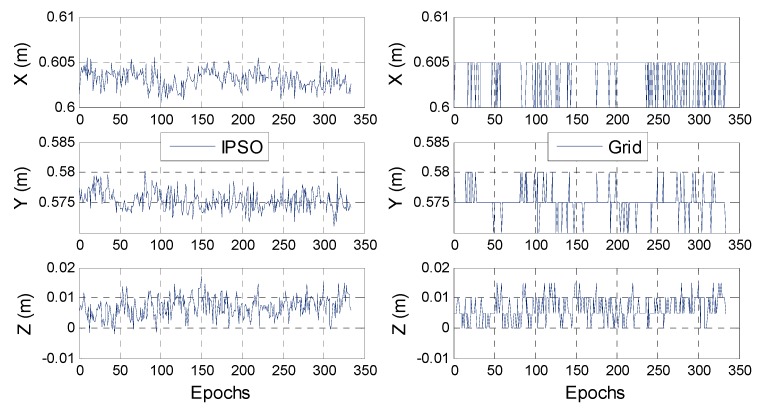
Final pseudolite positioning results with two different AFM searching methods.

Figure 7 shows the single-epoch positioning results for the IPSO and grid search methods. The two search results are in good agreement and all of the 3-D coordinates for each epoch fluctuated slightly within 3 cm near the true position, indicating that the IPSO search method has a good global search ability and does not appear to fall into the local optimal situation. In addition, the minimum resolution of the grid search result is 5 mm, whereas the IPSO can perform a more detailed search than the grid search, which is another significant advantage of IPSO.

**Figure 8:**
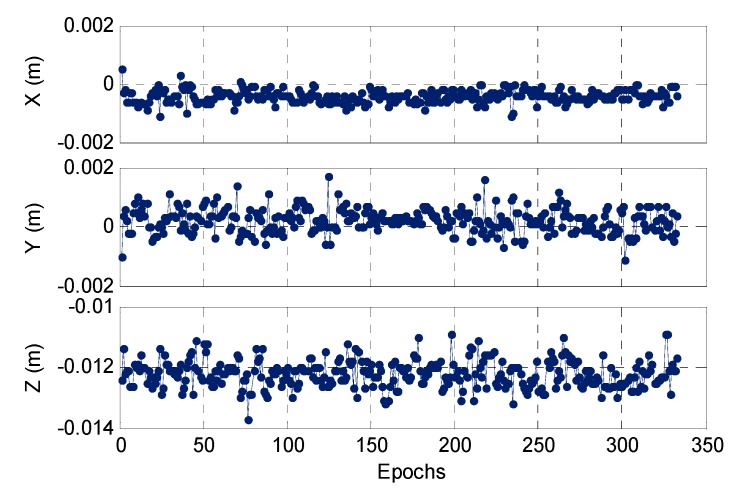
Differences in positioning results between AFM and LS methods.

As we obtained the optimal coordinate solution of the rover station, the corresponding DD ambiguity can be gained from the integer round (IR) method. Considering the DD ambiguity, we can obtain another coordinate solution using LS estimation. Theoretically, the AFM and LS methods are equivalent. The former is nonlinear, while the latter is a linear estimation method. For GNSS positioning, we can ignore the first-order linearization error, and thus, the positioning results using AFM and LS should be generally consistent. However, the indoor space is relatively small, and it can be expected that a coordinate linear error will exist when using the LS estimation method. Figure 8 shows the difference between the final positioning results obtained through the AFM and LS methods. These two methods have a 2 mm difference on the plane and an elevation difference of approximately 1.2 cm, which means that the first-order linear error can cause a cm-level positioning deviation for indoor pseudolite positioning.

The validity of the proposed AR method is verified by selecting four additional points for a further static test. The initial approximate coordinates, observation epoch number, pseudolite number, and position dilution of precision (PDOP) values of the four fixed points are shown in Table 3. The search space is set to 0.2 m × 0.2 m × 0.2 m, in which the initial approximate coordinates given are within the accuracy of 3 cm.

**Figure 9:**
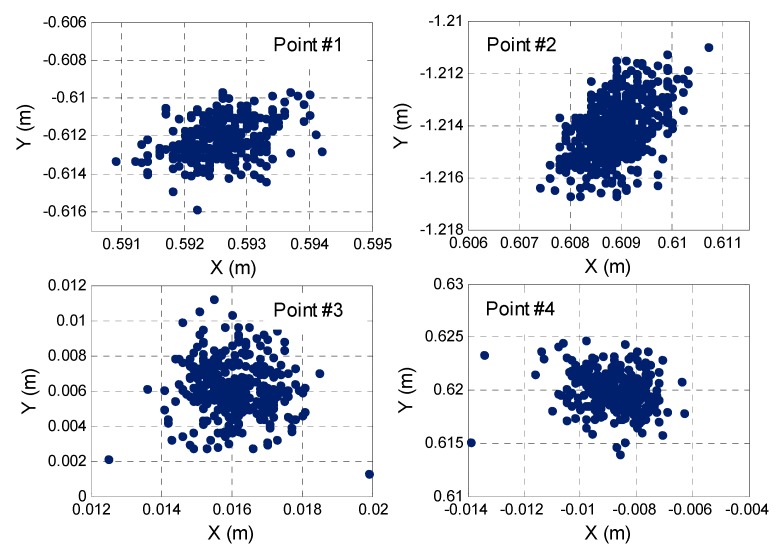
2-D positioning results with the static test.

Figure 9 shows the 2-D positioning results that correspond to the four fixed points. For the four different spatial distribution points, the IPSO method can search for the optimal solution with a good performance, further verifying the robustness of the IPSO search method. The pseudolites and rover station are in a static state under static observation, and thus, theoretically speaking, the search optimal coordinates of each epoch for every point should be consistent. Figure 9 shows that the search results of the four points are in a discrete distribution, with a standard deviation of about 0.005 m, which can be attributed to the noise error of the carrier phase observation and the effects of multipath errors.

#### 3.2.2. Kinematic Test

**Figure 10:**
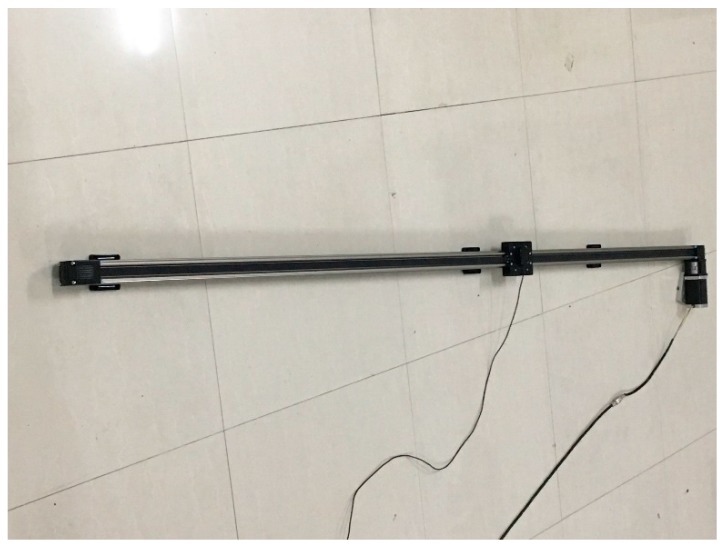
Fixed rail for the kinematic test.

A kinematic test is carried out to verify the validity of the proposed single-epoch AR method. The mobile car was placed in a fixed rail, as shown in Figure 10. The length of the rail is 2.2 m. During the entire experimental procedure, the mobile car’s moving speed is from slow to fast. The data sampling interval is 0.1 s. A total of five pseudolite signals can be captured without interruption. The initial coordinate on the first epoch is pre-given with an accuracy of 3 cm, and the subsequent epochs adopt the first method introduced in Section 2.3.2, because the data sampling rate is high and the speed of the mobile car is relatively slow. The corresponding AFM search space is set to 0.2 m × 0.2 m × 0.2 m.

**Figure 11:**
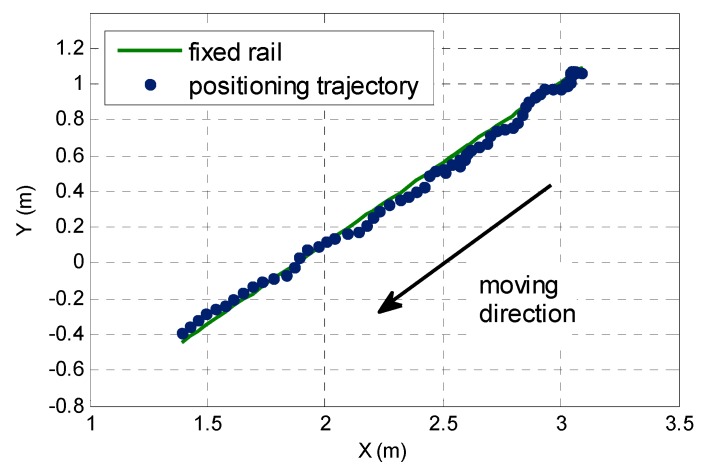
Kinematic trajectory based on the pseudolite positioning result.

Figure 11 shows the final pseudolite positioning results for this kinematic test. The overall positioning trajectory is relatively smooth. The relative deviation from the straight line of most points is within 5 cm. The calculated baseline length using the positioning results of the starting and stopping points is 2.224 m, with an actual rail length difference of 0.024 m, which means that the kinematic positioning precision of the indoor pseudolite can reach a cm-level using the proposed single-epoch AR method.

**Figure 12:**
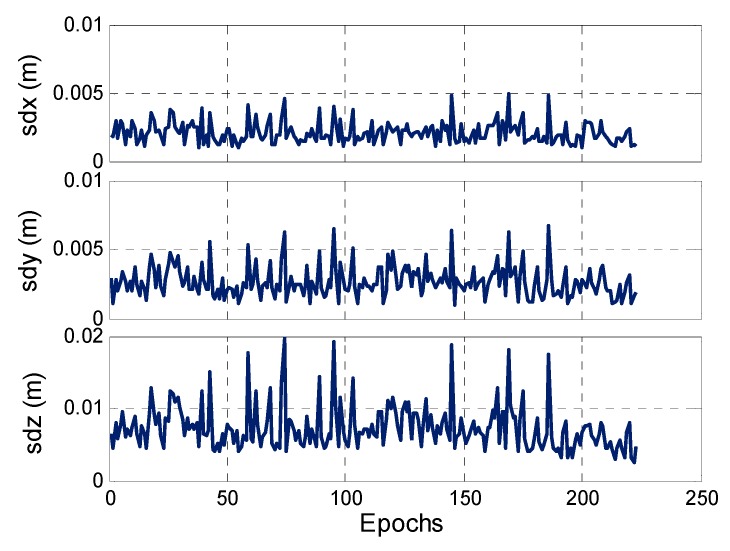
Kinematic positioning errors computed by LS method.

The wavelength of the GPS L1-band is 19 cm; that is, if the ambiguity is solved incorrectly with one cycle bias, it can cause cm- or even dm-level positioning errors. Generally, we can use the variances of the baseline parameters estimated using the LS method to validate the search optimal solution of the rover station during each epoch. Figure 12 shows the 3-D positioning error sequence of each epoch for this kinematic test. The 2-D positioning error is within 1 cm and the elevation direction is relatively large, that is, less than 2 cm, indicating that the solved DD ambiguity for every epoch is correct.

## 4. Conclusions

In this paper, we propose a well-suited AR method based on AFM for an indoor pseudolite system. This method can be applied to a low-cost single-frequency software receiver, and only requires a carrier phase observation, which can be realized with a single-epoch AR. This method is also nonlinear, which can effectively avoid the effect of the first-order linear truncation error. Considering the very low efficiency of conventional AFM, we adopt the IPSO algorithm to replace the conventional grid search method because it can significantly improve the AFM computational efficiency and has a more elaborate search ability. This study validates the reliability of the proposed AR method using indoor pseudolite observation data. As long as the initial approximate coordinates can be obtained accurately (better than 0.2 m), the proposed AR method exhibits a good performance. When considering a static observation, the standard variance of the positioning result is less than 0.005 m, and the kinematic observation using indoor pseudolite positioning, with the corrected ambiguity gained from our proposed method, can achieve a centimeter-level precision using a low-cost single-frequency software receiver.

Currently, some indoor positioning technologies like Ultra-WideBand (UWB) have been able to achieve a positioning accuracy better than 0.2 m. Therefore, in the near future it can be expected that the results of UWB positioning (or other indoor positioning technologies) can be used as the initial search coordinates of the proposed method in this paper.

## Figures and Tables

**Table 1:** The influence of initial coordinate bias (ICB) on the two different AR methods.

ICB (m)	LAMBDA Method (Ratio Threshold: 3)				Proposed AR Method	
	Best Candidate (✓/×)	Ratio	Success (Y/N)	Time (ms)	Success (Y/N)	Time (ms)
0.05	✓	4.6	Y	2	Y	23
0.10	✓	2.3	N	2	Y	22
0.15	×	1.6	N	2	Y	25
0.20	×	1.5	N	2	Y	26

**Table 2:** Comparison of AFM efficiency with two different searching methods (unit: s).

	IPSO	Grid		
		Search Step (m)		
**Search Space (m)**	/	0.01	0.005	0.001
0.1	0.0211	0.0154	0.1486	13.9678
0.2	0.0265	0.1166	0.8923	/
0.3	0.0343	0.3809	3.0126	/

**Table 3:** Static test with four other fixed points.

	Initial Coordinates	Total Epochs	Pseudolites Number	PDOP
Point #1	(0.6, −0.6, 0.01)	545	5	3.5
Point #2	(0.6, −1.2, 0.01)	536	4	4.1
Point #3	(0.0, 0.0, 0.01)	342	5	3.1
Point #4	(0.0, 0.6, 0.01)	298	5	3.2
